# Fermitin family homolog-2 (FERMT2) is highly expressed in human placental villi and modulates trophoblast invasion

**DOI:** 10.1186/s12861-018-0178-0

**Published:** 2018-11-01

**Authors:** Eiko Kawamura, Gina B. Hamilton, Ewa I. Miskiewicz, Daniel J. MacPhee

**Affiliations:** 1 Department of Veterinary Biomedical Sciences, Western College of Veterinary Medicine, University of Saskatchewan, 52 Campus Dr, University of Saskatchewan, Saskatoon, SK S7N 5B4 Canada; 2 Faculty of Medicine, Memorial University of Newfoundland, St. John’s, NL A1B 3V6 Canada; 3 One Reproductive Health Research Group, University of Saskatchewan, Saskatoon, SK S7N 5B4 Canada

**Keywords:** FERMT2, Kindlin-2, Integrins, Placenta, Trophoblast, Differentiation

## Abstract

**Background:**

Integrins are transmembrane receptors that mediate cell–extracellular matrix (ECM) and cell-cell adhesion and trophoblast cells undergo changes in integrin expression as they differentiate. However, the mechanism(s) of integrin activation leading to integrin-mediated signaling in trophoblast cell differentiation is unknown. The Fermitin family proteins are integrin activators that help mediate integrin-mediated signaling, but have never been studied in detail within the human placenta. Thus, we examined the spatiotemporal pattern of expression of Fermitin family homolog-2 (FERMT2) in human chorionic villi throughout gestation and its role in trophoblast-substrate adhesion and invasion.

**Methods:**

Placental villous tissue was obtained from patients undergoing elective terminations by dilatation and curettage at weeks 8–12 (*n* = 10), weeks 13–14 (*n* = 8), as well as from term deliveries at weeks 37–40 (*n* = 6). Tissues were fixed, processed and sections utilized for immunofluorescence analysis of FERMT2 expression during gestation. Additionally, HTR8-SVneo human trophoblast cells were transfected by electroporation with FERMT2-specific siRNAs or non-targeting siRNAs (control) and used in cell-substrate adhesion as well as invasion assays.

**Results:**

FERMT2 was more commonly expressed in the basal domain of villous cytotrophoblast cells and prominently localized around the periphery of individual extravillous trophoblast cells. siRNA-mediated knockdown of FERMT2 in HTR8-SVneo cells resulted in significantly decreased trophoblast-substrate attachment (*p* < 0.05) as well as significantly decreased trophoblast invasion (p < 0.05) relative to control cells.

**Conclusions:**

The detection of FERMT2 throughout extravillous trophoblast columns and the results of invasion assays demonstrated that this protein is likely an important regulator of integrin activation in extravillous cells to modulate migration and invasion.

## Introduction

In the human placenta, finger-like structures termed chorionic villi develop and become spatially segregated into floating and anchoring villi [1]. Floating villi are the majority of chorionic villi and are bathed in maternal blood while anchoring villi are involved in establishing and maintaining the fetal-maternal interface. During early placentation, two fundamental pathways of trophoblast differentiation take place in these villi. In the fusion pathway, polarized stem villous cytotrophoblast (CT) cells in floating villi proliferate, while sitting on a villous basement membrane, and daughter cells then differentiate and fuse with existing and overlying syncytiotrophoblast to maintain the multi-nucleated layer [2]. Changes in CT morphology over gestation and the regulation of syncytialization have been described in detail elsewhere [3–10].

In the invasion pathway, polarized stem CT cells in anchoring villi tips migrate off the villous basement membrane, penetrate through the syncytiotrophoblast to form columns of non-polarized extravillous trophoblast (EVT) cells which connect the embryo to the uterine wall [11, 12]. The formation of anchoring villi is accompanied by changes in the synthesis and spatial distribution of extracellular matrix (ECM) proteins and concurrent alterations in the spatiotemporal expression of ECM-binding integrin receptors within EVT [13–16]. The differentiation and net invasiveness of trophoblast cells during early placental development was shown to be determined, at least in part, by the regulation of integrin-mediated adhesion mechanisms [14, 17, 18]. Despite this knowledge, the mechanism(s) of integrin activation leading to integrin-mediated signaling in trophoblast cell differentiation is still unknown and requires study as abnormalities in the development of the CT, syncytiotrophoblast, and invasive EVT contribute to the development of diseases during pregnancy [1, 12]. For example, shallow trophoblast invasion has been associated with preeclampsia and this gestational trophoblast disease has also been correlated with an abnormal expression of integrins ITGA6/B4 and ITGA1/B1 [12, 19].

The Kindlin or Fermitin (FERMT) family is a group of adapter or scaffold proteins that are critical for signaling to and from membrane-spanning integrin adhesion receptors [20, 21]. The original nomenclature of the family originates from a rare congenital skin disease in humans named Kindler syndrome caused by mutations in the Kindlin-1 (Fermitin family homolog 1; *FERMT1*) gene [22–24]. The FERMT proteins (FERMTs) all possess a 4.1 protein, ezrin, radixin, moesin (FERM) domain that now defines their family name. FERMT1 expression is mainly restricted to epithelia, FERMT2 is ubiquitously expressed, and FERMT3 expression is mostly confined to the hematopoietic system [25–27].

FERMTs, in concert with talin, can activate integrins by eliciting conformational changes in these receptors leading to increased integrin affinity for their extracellular ligands and regulating integrin-mediated adhesion and signaling [28–31]. Once integrins are activated by FERMTs, FERMTs can stay at cell-ECM adhesion sites and link integrins to the cytoskeleton to elicit changes in cell behavior [20, 21]. The interaction of FERMTs with actin regulatory proteins such as integrin-linked kinase (ILK), migfilin (FBLIM1), and focal adhesion kinase (PTK2) underlie this signaling [21, 32, 33]. We have previously demonstrated that ILK and PTK2 are highly expressed in CT and EVT [34, 35]. Furthermore, ILK regulates trophoblast migration and syncytialization [35–37]; however, the expression of FERMTs in the human placenta is unknown. The objective of our study was to determine the expression of FERMT2 in human chorionic villi throughout gestation and determine the role of this integrin activator in trophoblast-substrate adhesion and invasion.

## Methods

### Tissue collection

Placental tissue was obtained from elective terminations, following dilatation and curettage, at weeks 8–12 (*n* = 10), weeks 13–14 (*n* = 8) as well as from term deliveries at weeks 37–40 (*n* = 6). Tissues were collected in sterile phosphate-buffered saline (PBS; pH 7.4) and transported to the laboratory within 10 min of collection. All tissue samples were extensively washed, dissected in cold PBS, processed and fixed as previously described [35]. Tissue processing, embedding and sectioning were conducted by the Histology Unit of the Faculty of Medicine, Memorial University of Newfoundland. Two 5 μm thick serial tissue sections were mounted per glass slide so one section could serve as a negative control for experiments.

### Immunofluorescence analysis

Tissue sections were dewaxed in xylene and rehydrated in a descending series of ethanol followed by a PBS wash. Heat-induced epitope retrieval was performed in a 10 mM sodium citrate solution (pH 6.0) as previously described in detail [38]. Subsequently, tissue sections were incubated with 0.1% Trypsin/PBS (Cat # T7168; Sigma Aldrich) for 10 min at room temperature and then washed in PBS [35].

Tissue sections were blocked in 5% normal goat serum/1% horse serum/1% fetal bovine serum in PBS to prevent non-specific antibody binding. Sections were then incubated overnight at 4 °C in appropriate primary antiserum (Table 1) or with affinity purified control immunoglobulin (IgG) of the appropriate species used at the same concentration as the primary antiserum. All tissue sections were then washed with PBS and incubated with appropriate secondary antiserum (Table 1), diluted in blocking solution, for 1 h at room temperature. When co-immunofluorescence analysis was conducted, sections were then washed in PBS and re-blocked for 1 h followed by addition of the next appropriate primary antiserum (Table 1) or control IgG to tissue sections and incubation overnight at 4 °C. After incubation in secondary antiserum and washes in PBS containing 0.02% Tween-20, tissue sections were mounted in Vectashield containing 4′,6-diamidino-2-phenylindole (DAPI; Cat # H-1200; Vector Laboratories) and sealed with nail polish. All immunofluorescence experiments were repeated at least 6 times. Images were acquired with a Leica DM-IRE2 inverted microscope (Leica Microsystems), equipped for epi-fluorescence illumination, using a plan apochromat 40X/NA 0.85 objective lens and a Retiga EXi CCD camera (Qimaging) or an Olympus BX51 microscope (Olympus) using a UplanFL N 40X/NA 0.75 objective lens and an Olympus DP70 colour CCD camera. Openlab Image Analysis software (Perkin Elmer) or DP Controller/ DP Manager software (Olympus) was utilized for image capture and processing.

**Table 1:** Antisera utilized for immunofluorescence and immunoblot analyses

Antisera	Method/Dilution Used	Company	Catalogue #
Mouse anti-FERMT2 Clone 3A3	IF: 1:50 IB: 1:4000	EMD Millipore, Etobicoke, ON, CA	MAB2617
Rabbit anti-ITGA5	IF: 1:250	EMD Millipore	AB1928
Rabbit anti-ITGA6	IF: 1:100	Sigma Aldrich, Oakville, ON, CA	HPA012696
Rabbit anti-VWF	IF: 1:200	EMD Millipore	AB7356
Rabbit anti-CDH1	IF: 1:50	Sigma Aldrich	HPA004812
Mouse anti-FERMT1 Clone KN-4	IB: 1:2000	Sigma Aldrich	SAB4200465
Mouse anti-TUBA Clone DM1A	IB: 1:20000	Sigma Aldrich	T6199
Sheep anti-Rabbit FITC	IF: 1:250	Sigma Aldrich	F7512
Donkey anti-Mouse RRX	IF: 1:150	Jackson ImmunoResearch, West Grove, PA, US	715–295-150
ChromPure Mouse IgG	IF: **	Jackson ImmunoResearch	015–000-003
ChromPure Rabbit IgG	IF: **	Jackson ImmunoResearch	011–000-003
Goat anti-Mouse HRP	IB: 1:10000	Promega, Madison, WI, USA	W4021

*IF*: Immunofluorescence, *IB*: Immunoblot, *FITC*: Fluorescein isothiocyanate, *RRX*: Rhodamine-Red-X, *HRP*: horseradish peroxidase. **Matched to concentration of primary antisera utilized

### Cell culture

The human trophoblast cell line HTR8-SVneo was obtained from Dr. Charles Graham (Queens University, Kingston, ON, Canada). The cell line was derived from primary human villous explants, is invasive and non-tumourigenic and demonstrates an EVT immunological and biological phenotype [39–45]. Cells were cultured at 37 °C under 5% CO_2_ in air and maintained in RPMI-1640 (Cat. #11875–093, Life Technologies) supplemented with 10% fetal bovine serum (FBS) (Cat. #12483–020; Life Technologies) and 100 Units penicillin/100 μg streptomycin (Cat. #15140–122; Life Technologies).

### siRNA transfection

HTR8-SVneo cells (1 × 10^6^ cells) were transfected with 50 nM FERMT2-specific small interfering ribonucleic acids (siRNAs) by electroporation using a Neon Transfection System (Life Technologies) according to the manufacturer’s detailed instructions. To achieve effective depletion, a mixture of four siRNAs specific to human FERMT2 (SMARTpool ON-TARGETplus, Cat. #L-012753-00-0005, Dharmacon) was used, while a cocktail of four non-targeting siRNAs served as a negative control (ON-TARGETplus Non-targeting pool, Cat. #D-00180-10-05, Dharmacon). Control siRNAs were used at the same concentrations as the FERMT2 targeting siRNAs. Transfected cells were grown in RPMI-1640 media supplemented with 10% FBS, seeded in 6-well or 96-well tissue culture plates, and cultured as described above.

### Immunoblot analysis

Transfected HTR8-SVneo cells were lysed in 1 x sodium dodecyl sulphate (SDS) gel loading buffer (50 mM Tris-HCl, pH 6.8, 2% SDS, 10% glycerol, 100 mM 2-mercaptoethanol) without dyes and immediately boiled for 5 min. Protein concentrations were determined by the Bradford Assay [46] using the Bio-Rad protein assay dye reagent (Cat. #500–0006; Bio-Rad Laboratories). Protein lysates (10 μg protein/lane) were separated by SDS-polyacrylamide gel electrophoresis (PAGE) and electroblotted to 0.2 μm nitrocellulose membranes (Cat #: 162–0097; Bio-Rad). After blocking the membranes with 5% skim milk in Tris-buffered saline Tween-20 (TBST; 20 mM Tris, 137 mM NaCl, and 0.1% Tween-20, pH 7.6) for 1 h, membranes were incubated with appropriate specific primary antibodies (Table 1). Immunoblots were then washed with TBST followed by incubation in horseradish peroxidase-conjugated secondary antiserum (Table 1) for 1 h. The immunoblots were again washed with TBST and protein-antisera complexes detected using the Pierce SuperSignal West Pico chemiluminescent substrate detection system (Cat. # 34080; ThermoFisher Scientific). Multiple exposures were acquired using a Bio-Rad ChemiDoc MP digital imaging system. Membranes were subsequently probed for Tubulin (TUBA) expression, which served as a loading control.

### MTT assays

After transfection, 2.7 × 10^3^ cells were plated within each well of a 96-well tissue culture plate in triplicates or quadruplicates and 3-(4,5-dimethylthiazol-2-yl)-2,5-diphenyltetrazolium bromide (MTT) assays conducted based on the Roche Molecular Biochemicals protocol according to Dahlgren et al. [47]. Ten microlitres of 1 mg/ml MTT (Sigma Aldrich) in PBS was added to each well. After 4 h of incubation, cells were lysed with 10% SDS and 0.01 M HCl overnight. OD_550_ and OD_690_ were then measured with a Spectra Max 190 spectrophotometer (Molecular Devices). To determine degrees of cell proliferation, background values at OD_690_ were subtracted from values at OD_550_. Experiments were conducted four times. Values were normalized to the non-targeting siRNA treatment group.

### Invasion assays

Invasion assays were performed using an QCM ECMatrix cell invasion assay kit (Cat. #ECM554, EMD Millipore) according to the manufacturer’s instructions. Briefly, cells were serum starved overnight, trypsinized and 1.25 × 10^5^ cells seeded into each upper chamber containing serum-free culture media while the bottom chamber was filled with culture media supplemented with 10% FBS to serve as an attractant. After 48 h, cells that had invaded into the lower chamber were detached, lysed, stained with CyQuant GR® dye, which binds nucleic acids, and the fluorescence intensity measured with a fluorescence microplate reader (FLx800, Bio-Tek Instruments Inc.). Assays were conducted in duplicate or triplicate and experiments were repeated four times. Values were normalized to the non-targeting siRNA treatment group.

### Adhesion assays

Adhesion assays were performed according to Humphries [48] with minor modifications. Briefly, 48 h after transfection with siRNAs, cells were trypsinized and 5 × 10^4^ cells were plated in each well of a 96-well plate in triplicates or quadruplicates. Cells were left to adhere to the plastic surface for 15 min, and the dishes disturbed by tapping before fixing adhered cells with 4% paraformaldehyde in PBS. Adhered cells were stained with 0.1% crystal violet in 20% methanol. After washing with distilled water, crystal violet was solubilized in 10% acetic acid and OD_570_ was measured with a Spectra Max 190 spectrophotometer. Experiments were conducted four times and values were normalized to the non-targeting siRNA treatment group.

### Data analysis

Densitometric analysis on immunoblot data was performed using Image Lab software (Bio-Rad). Statistical analysis was performed with GraphPad Prism version 5.01 (GraphPad) or Microsoft Excel (Microsoft). Statistical significances for MTT, invasion and adhesion assays, as well as densitometric analysis were analyzed with paired two-tailed t-tests. Statistical significance was ascribed to a *P* value < 0.05.

## Results

### Expression of FERMT2 within placental villi

**Fig. 1:**
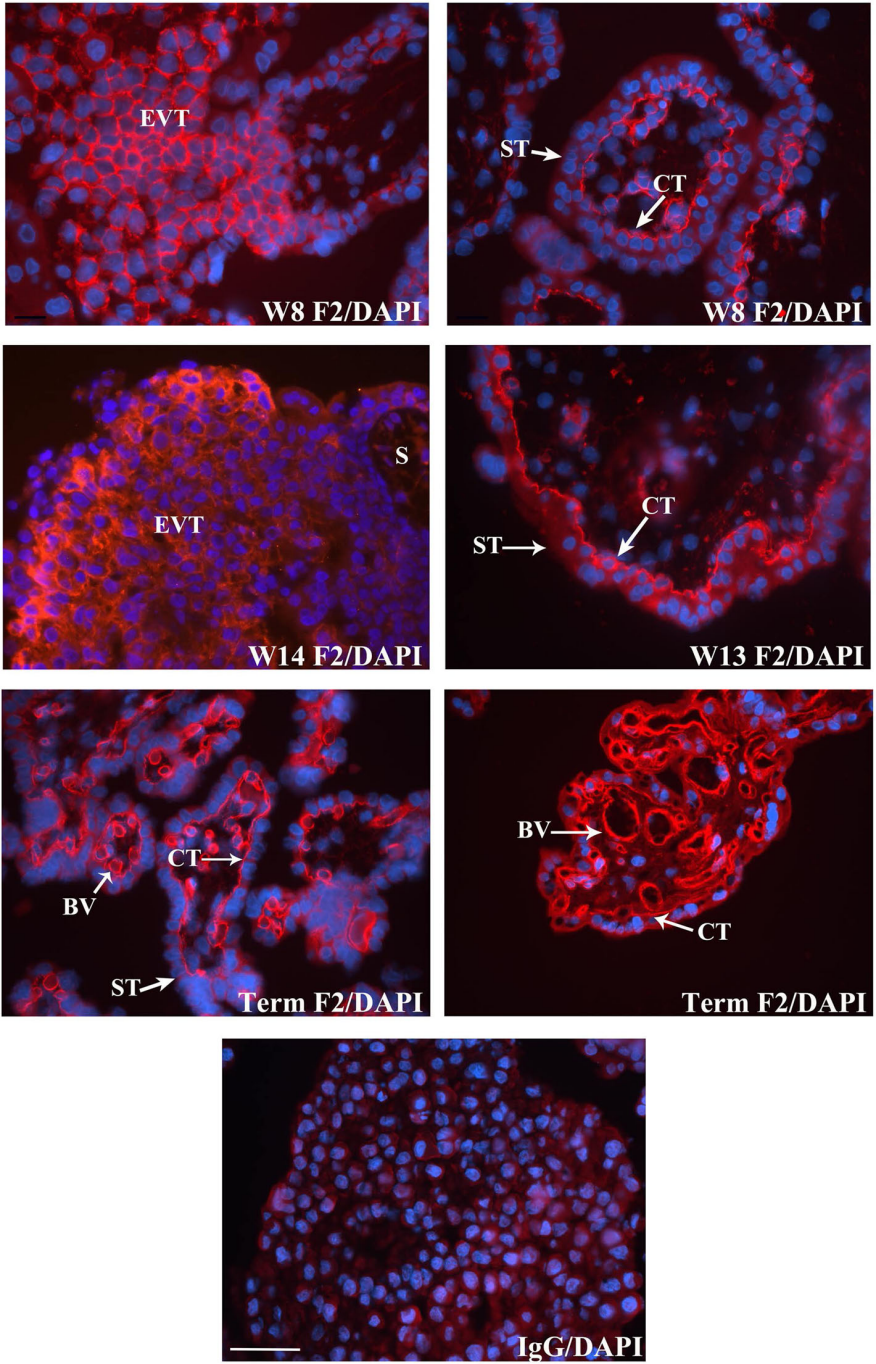
Immunofluorescence detection of FERMT2 (F2) in human placental tissue at week (W) 8, 13, 14, and term pregnancy. Representative images are shown. FERMT2 was continuously expressed in stem villous cytotrophoblast (CT) of floating villi throughout gestation and detected in proximal and distal extravillous trophoblast (EVT) of anchoring villi during the first and second trimester. FERMT2 was also detected in stromal mesenchyme (S) and putative developing blood vessels (BV), particularly at term pregnancy. IgG: mouse immunoglobulin used in place of primary antiserum. ST: syncytiotrophoblast. Nuclei were stained with DAPI. Scale bar = 50 μm

FERMT2 was highly expressed in chorionic villi throughout gestation (Fig. 1). Specifically, FERMT2 was immunolocalized to CT of floating villi and EVT of anchoring villi from the first and second trimester. Spatially, FERMT2 was detected in membrane-associated regions around some CT cells, but more commonly in the basal domain of the cells associated with the basement membrane (Fig. 1). In EVT, FERMT2 was prominently localized around the periphery of individual trophoblast cells and detected in apparent endothelial cells of developing villous blood vessels throughout gestation, including mesenchyme immediately surrounding the vessels at term pregnancy (Fig. 1). At term, FERMT2 was immunolocalized to the very thin CT of chorionic villi.

**Fig. 2:**
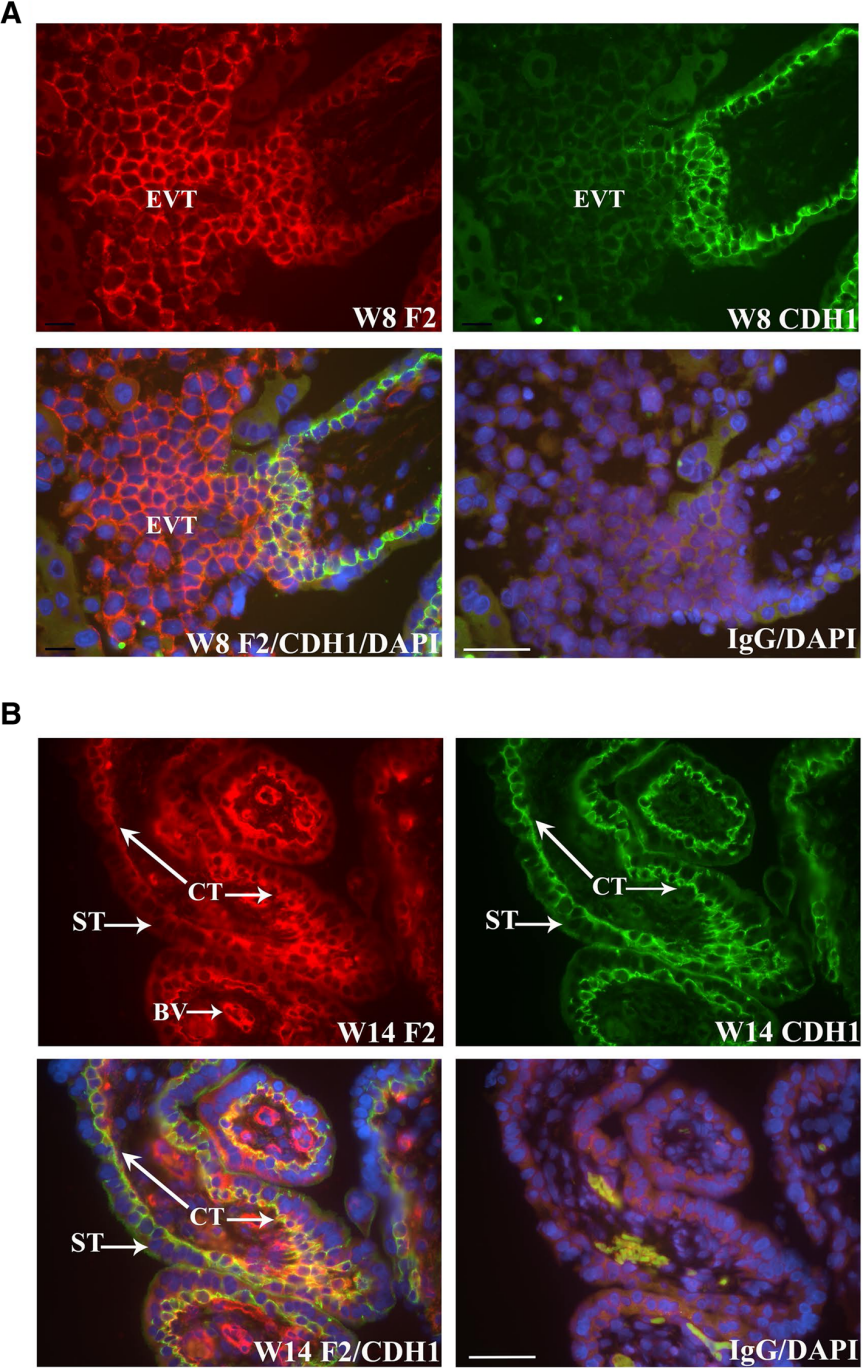
Co-immunofluorescence analysis of FERMT2 (F2) and CDH1 expression in human placental tissue during the first (**a**) and second (**b**) trimester. Representative images from week (W) 8 and W14 are shown. A) Co-immunolocalization of FERMT2 and CDH1 was observed in the most proximal portions of extravillous trophoblast columns (EVT). B) Marked co-immunolocalization was also noted in villous cytotrophoblast (CT). IgG: mouse and rabbit immunoglobulins used in place of primary antisera. BV: blood vessel; ST: syncytiotrophoblast. Nuclei were stained with DAPI. Scale bar = 50 μm

**Fig. 3:**
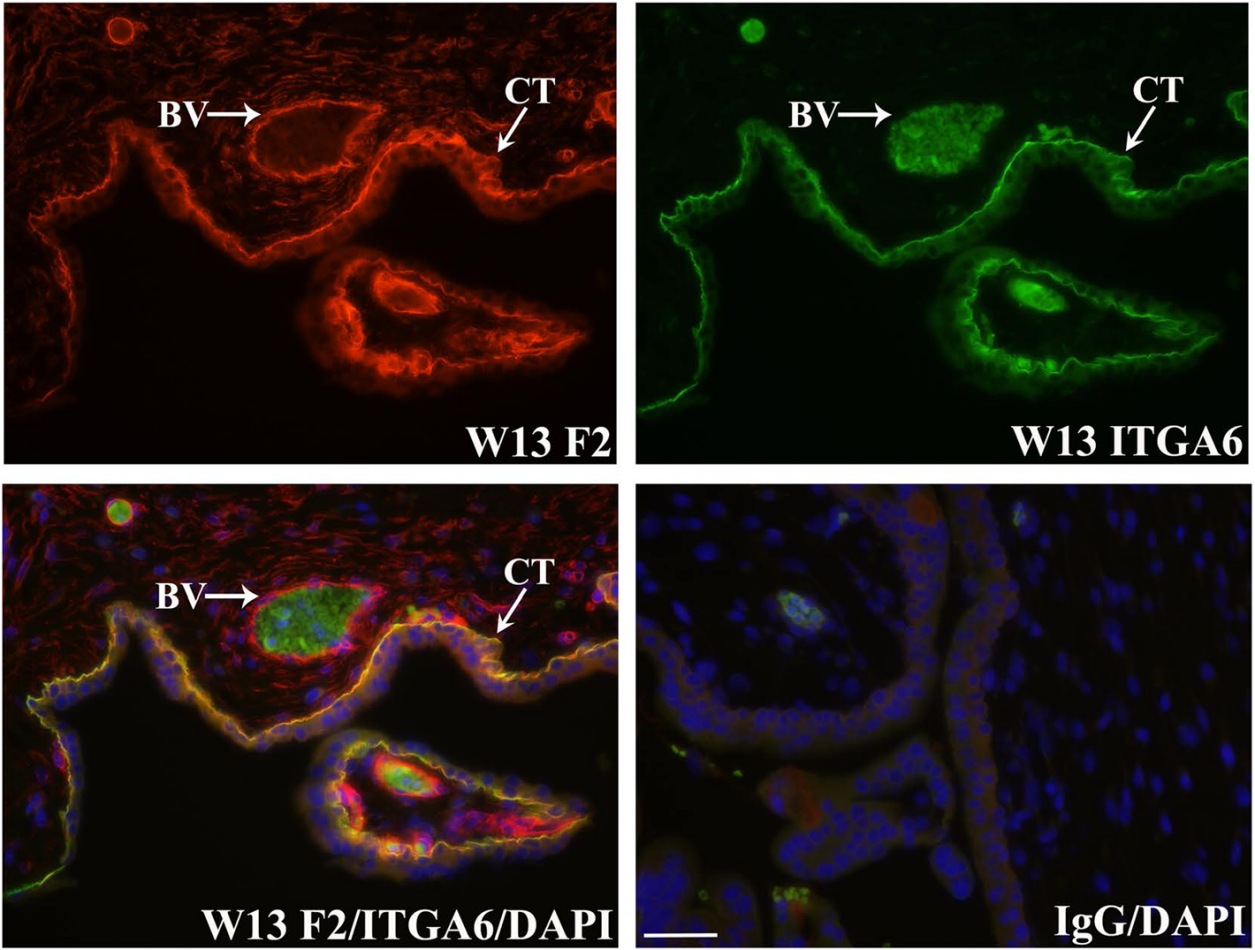
Co-immunolocalization of FERMT2 (F2) with ITGA6 in human placental tissue. Representative images at week (W) 13 of gestation are shown. FERMT2 was readily expressed with ITGA6 in the basal domain of the villous cytotrophoblast (CT) cells associated with the basement membrane. IgG: mouse and rabbit immunoglobulins used in place of primary antisera. BV: blood vessel. Nuclei were stained with DAPI. Scale bar = 50 μm

**Fig. 4:**
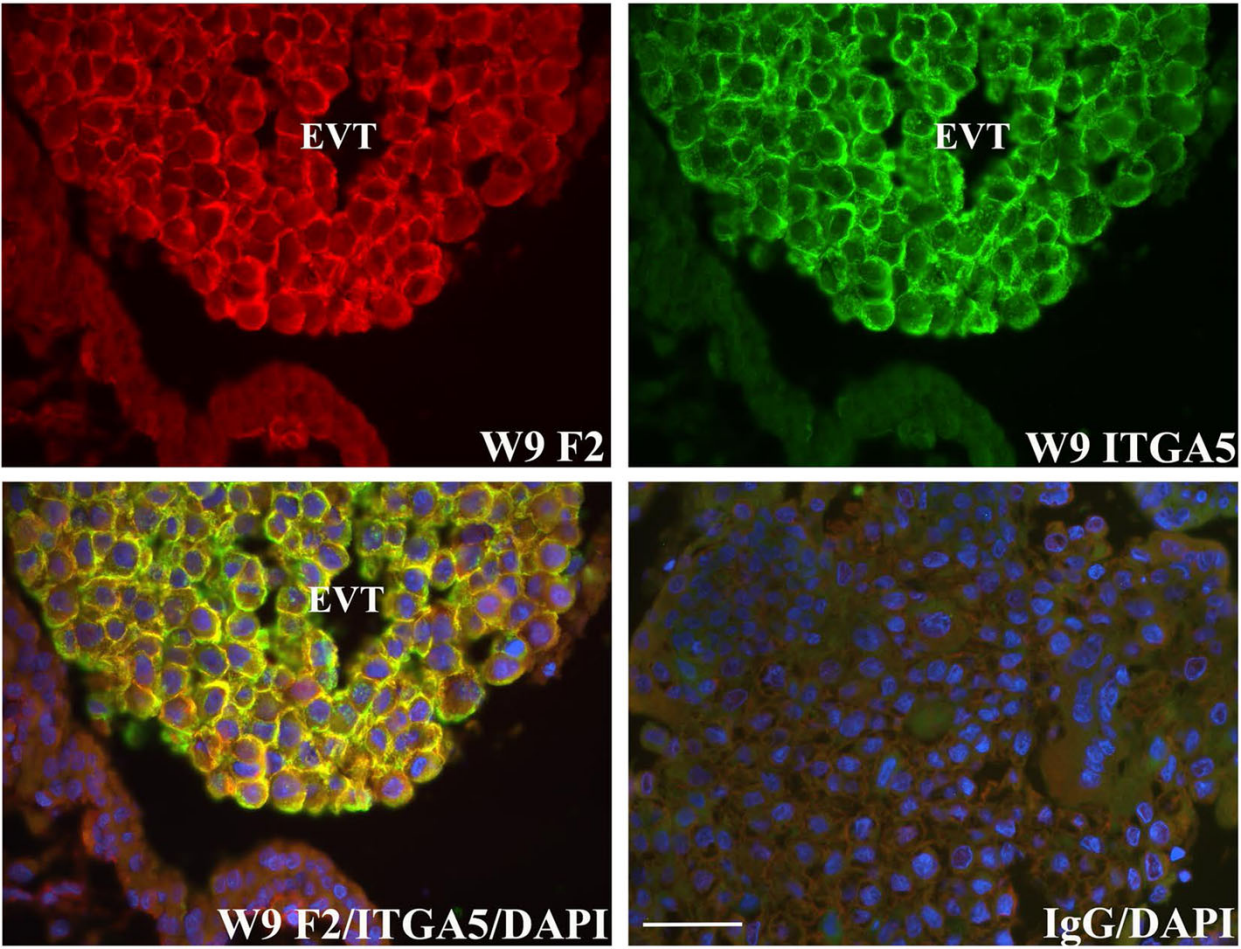
Co-immunofluorescence detection of FERMT2 (F2) with ITGA5 in human placental tissue. Representative images at week (W) 9 of gestation are shown. FERMT2 was intensely co-expressed with ITGA5 in more distal portions of extravillous trophoblast (EVT). IgG: mouse and rabbit immunoglobulins used in place of primary antisera. Nuclei were stained with DAPI. Scale bar = 50 μm

**Fig. 5:**
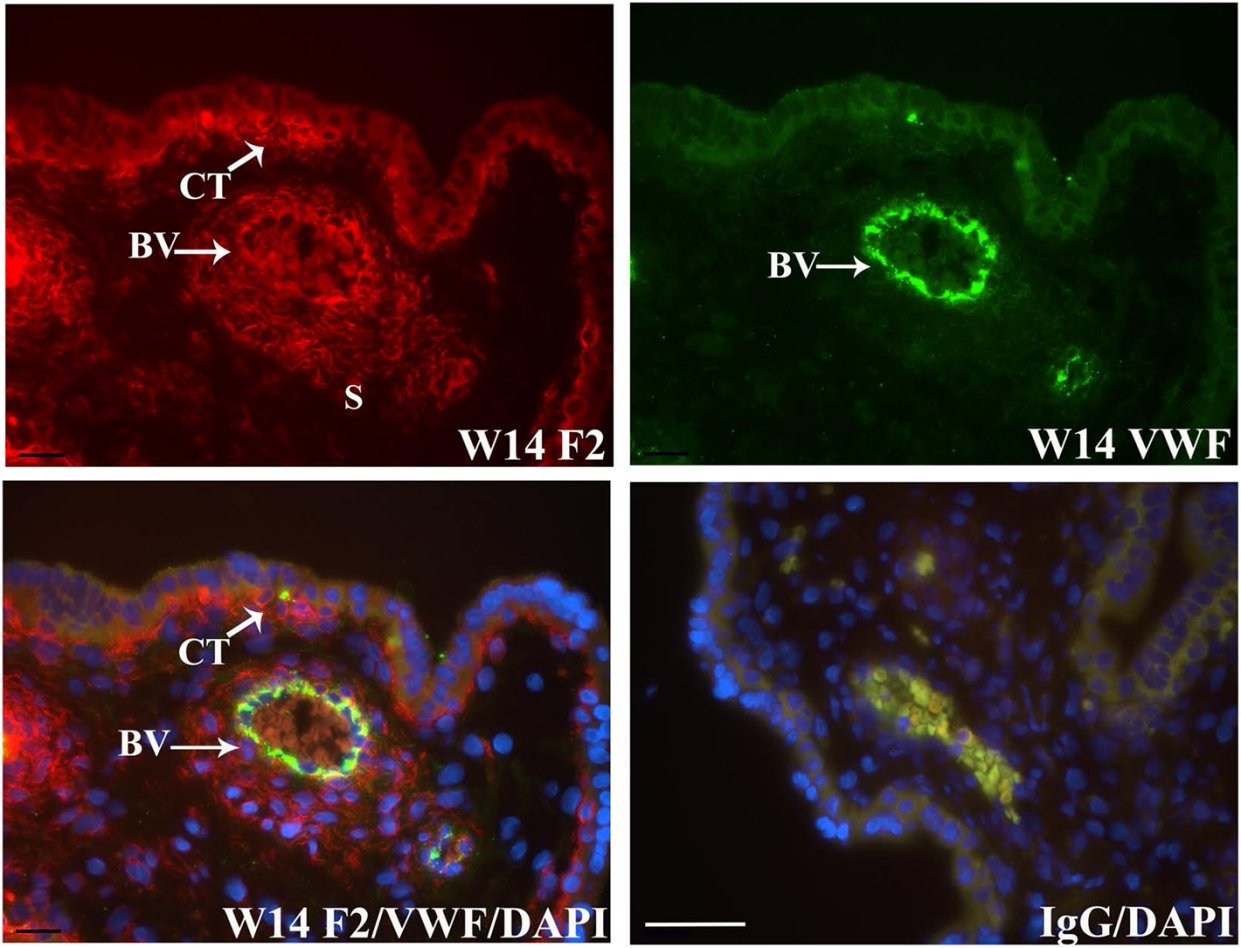
Co-immunofluorescence analysis of FERMT2 (F2) with von Willebrand Factor (VWF) in human placental tissue. Representative images are shown from week (W) 14 of gestation. FERMT2 and VWF were co-expressed in developing endothelial cells, but FERMT2 was also highly detectable in stromal mesenchyme around the developing blood vessels (BV). IgG: mouse and rabbit immunoglobulins used in place of primary antisera. CT: cytotrophoblast. S: stromal mesenchyme. Nuclei were stained with DAPI. Scale bar = 50 μm

To verify that FERMT2 localized to CT and EVT, co-immunofluorescence analysis was conducted with E-cadherin (CDH1), ITGA6, or ITGA5-specific antisera. CDH1 is highly detected at points of CT cell-cell contact and in EVT of proximal anchoring villi [9, 10]. FERMT2 readily co-localized with CDH1 in CT and proximal EVT of trophoblast columns (Fig. 2). Further analysis also showed that FERMT2 was co-expressed with ITGA6 in the basal domains of CT (Fig. 3) and in the proximal EVT of trophoblast columns (data not shown). In contrast, FERMT2 co-localized with ITGA5 in the more distal EVT of anchoring villi (Fig. 4). FERMT2 was also detected in endothelial cells of developing blood vessels in floating villi throughout gestation identified by co-localization with von Willebrand Factor (VWF) in these cells (Fig. 5).

### Investigation of the role of FERMT2 in trophoblast adhesion and invasion

**Fig. 6:**
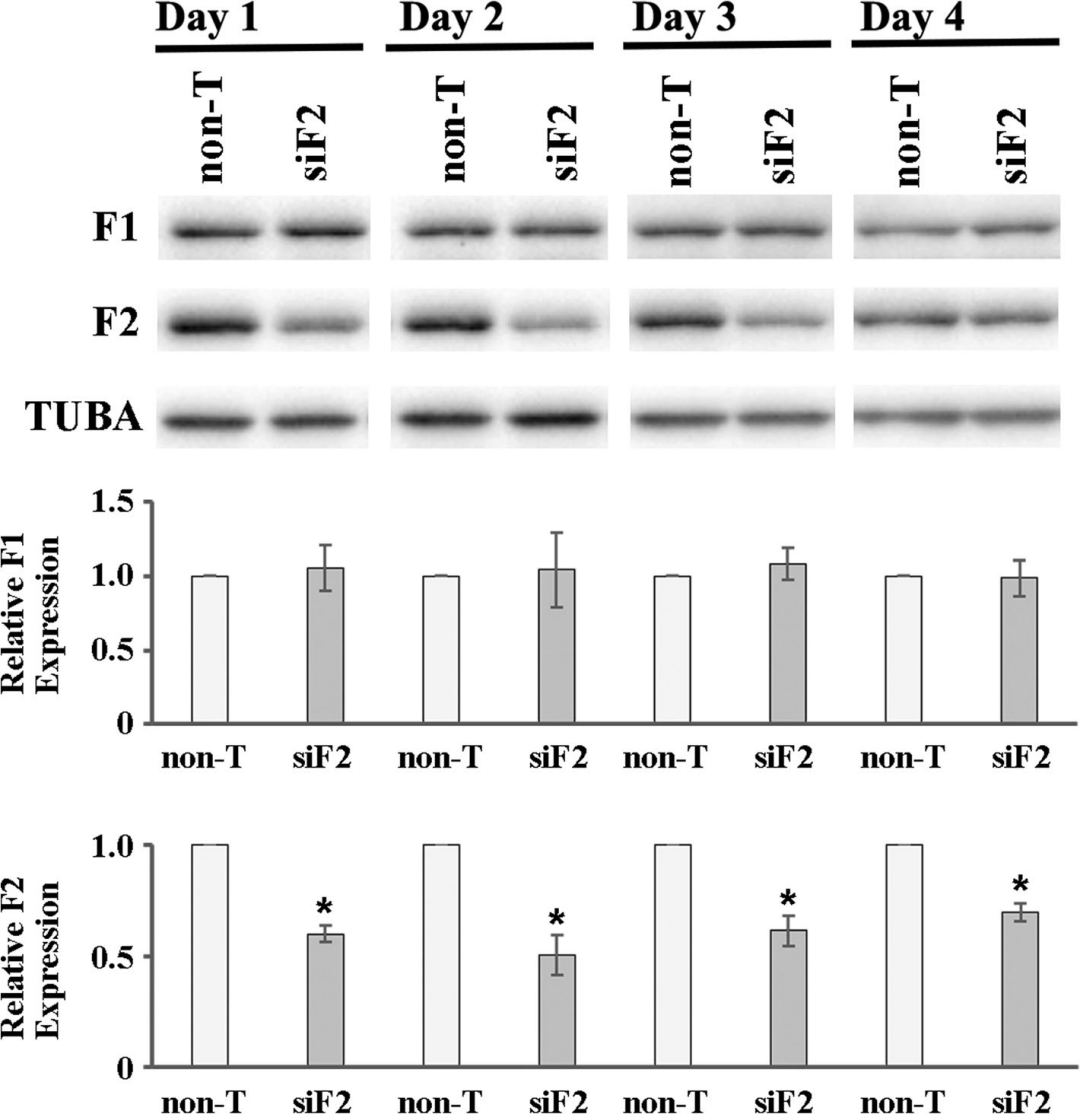
Confirmation of FERMT2 depletion after FERMT2-specific siRNA targeting in HTR8-SVneo cells. Following transfection of HTR8-SVneo cells with FERMT2-specific siRNAs (siF2) or non-targeting siRNAs (non-T), cell lysates were collected from 1 to 4 days post-transfection and targeted protein depletion established by immunoblot analysis. Blots were probed with FERMT1 (F1), FERMT2 (F2) or Tubulin (TUBA)–specific antisera. Tubulin expression served as a loading control. Representative immunoblots are shown. For densitometric analysis, the relative expression of F1 and F2 was calculated by normalizing values for FERMT2-depleted cells to corresponding non-T controls. Any differences in sample loading were equalized using corresponding TUBA densitometric values. The densitometric analysis shown is from four independent experiments. F2 expression was suppressed upon siF2 treatment (t-test, *p < 0.002). The expression of F1 was not significantly affected by F2 siRNA treatment confirming the specificity of the siRNAs

To investigate the role of FERMT2 in trophoblast cell adhesion and invasion, FERMT2 protein expression was depleted in the immortalized normal human HTR8-SVneo cell line using FERMT2-specific siRNAs. FERMT2 protein expression levels were then examined by immunoblot analysis from day 1 to day 4 post-transfection. A clear reduction in FERMT2 protein levels was observed by day 1 post-transfection after specific siRNA targeting, and also markedly reduced 2 and 3 days post-transfection, but on occasions did recover slightly on day 4 (Fig. 6). Immunoblot analysis was always used to verify FERMT2 expression depletion during experiments. Furthermore, the expression of FERMT1 was not affected by FERMT2 siRNA treatment, confirming the specificity of the siRNAs. This also indicated that there was likely no compensatory upregulation of FERMT1 when FERMT2 was depleted in HTR8-SVneo trophoblast cells.

**Fig. 7:**
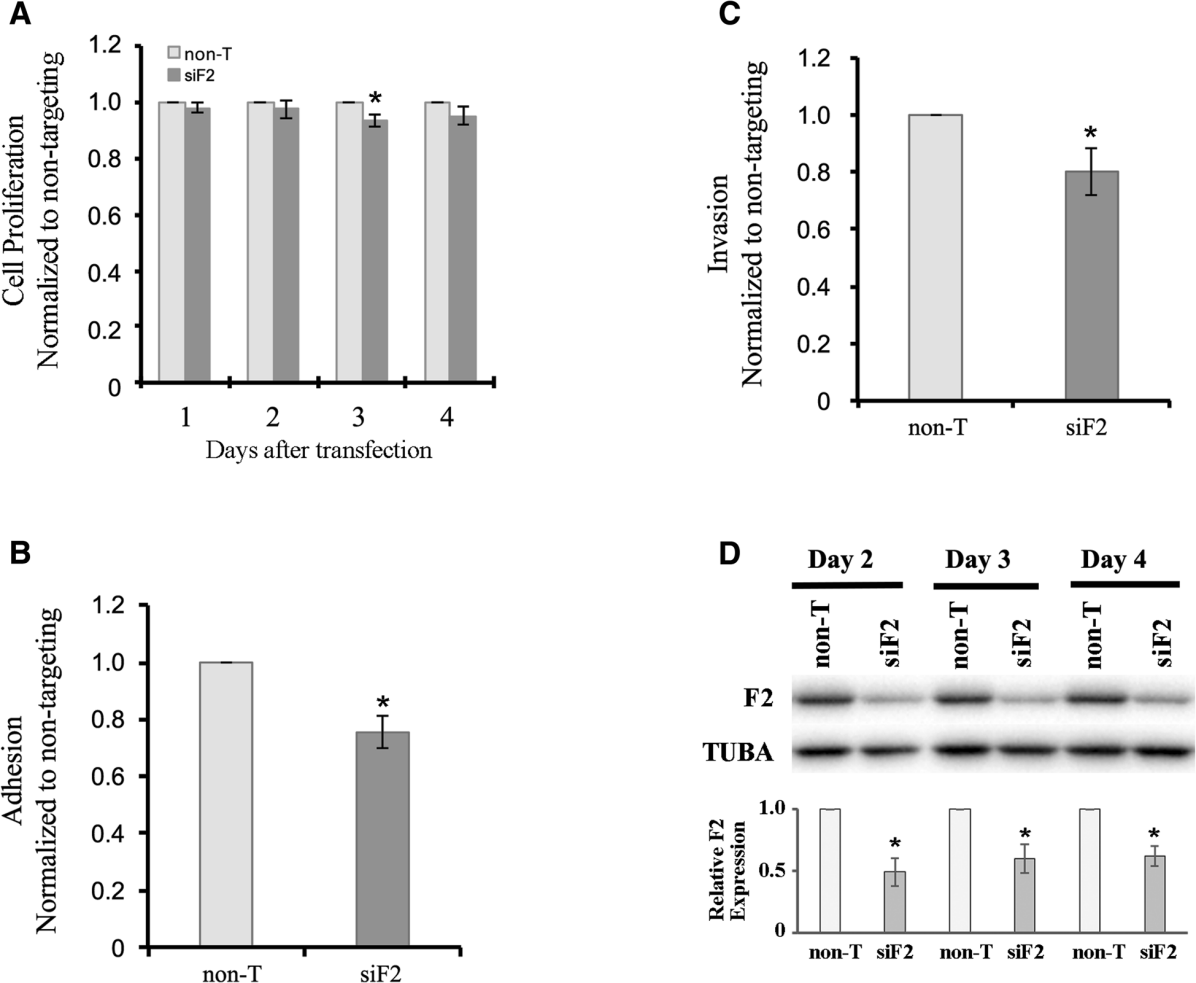
Characterization of the role of FERMT2 in trophoblast cell-substrate adhesion and cell invasion. **a**) MTT assays for HTR8-SVneo cell viability 1–4 days post-transfection with FERMT2 siRNA (siF2) or non-targeting control siRNAs (non-T). HTR8-SVneo cell proliferation was slightly, but significantly reduced on day 3 compared to control cells (*, *p* < 0.05). Data shown are from four independent experiments and values were normalized to the non-T treatment group. **b**) Adhesion assays after transfection of HTR8-SVneo cells with siF2 or non-T siRNAs. FERMT2 knockdown significantly reduced cell adhesion compared to control cells (*, p < 0.05). Data shown are from four independent experiments and values were normalized to the non-T treatment group. **c**) Invasion assays after transfection of HTR8-SVneo cells with siF2 or non-T siRNAs. FERMT2 knockdown significantly reduced trophoblast cell invasion compared to control cells (*, p < 0.05). Data shown are from four independent experiments and values were normalized to the non-T treatment group. **d**) Immunoblot analysis confirming FERMT2 depletion in HTR8-SVneo cells for 4 days following transfection with siF2. Tubulin (TUBA) expression was used as a loading control. Representative immunoblots are shown. For densitometric analysis, the relative expression of FERMT2 (F2) was calculated by normalizing values for FERMT2-depleted cells to corresponding non-T controls. Any differences in sample loading were equalized using corresponding TUBA densitometric values. The densitometric analysis shown is from four independent experiments. F2 expression was significantly suppressed upon siF2 treatment (t-test, *p < 0.01)

After transfection with FERMT2-specific or non-targeting control siRNAs, HTR8-SVneo cell viability was measured by MTT assays every 24 h from day 1 to day 4 post-transfection (Fig. 7). Cell proliferation rates were not significantly different between FERMT2-depleted cells and non-targeting siRNA control cells at day 1, 2 and 4 post-transfection. However, HTR8-SVneo cell proliferation was slightly but significantly reduced on day 3 (*p* = 0.013, paired t-test; Fig. 7a).

To examine if FERMT2 had a role in HTR8-SVneo cell-substrate adhesion, adhesion assays were performed two days after siRNA transfection. Trypsinized cells were left to adhere to the surface of plastic culture dishes and cell adhesion was measured by staining adhered cells with crystal violet. When FERMT2 protein expression was suppressed, cell adhesion was reduced approximately 25% compared to control cells (*p* = 0.025, paired t-test; Fig. 7b).

Since one of the characteristics of trophoblast cells is their ability to invade the maternal uterine wall, we determined if FERMT2 was necessary for cell invasion. Following siRNA transfection, HTR8-SVneo cells were serum starved and invasion assays were performed. Invasion of FERMT2-depleted cells was significantly reduced by approximately 20% (*p* = 0.018, paired t-test) compared to non-targeting siRNA treated cells (Fig. 7c). Marked depletion of FERMT2 protein expression was confirmed throughout the time period invasion assays were performed (Fig. 7d).

## Discussion

For the fusion and invasion pathways of trophoblast differentiation to be accomplished, trophoblast cells must alter cell-cell and/or cell-ECM adhesion interactions and many different signaling protein families are involved [1, 49, 50]. Integrin transmembrane protein receptors have important roles to play in regulating these adhesion processes as the differentiation and net invasiveness of trophoblast cells during early placental development was shown to be determined, at least in part, by the regulation of integrin-mediated adhesion mechanisms [14, 17, 18]. The FERMTs are a family of adapter or scaffold proteins that are critical for activating signaling to and from membrane-spanning integrin adhesion receptors [20, 21]; however, these proteins have never been characterized in the human placenta. Thus, we determined the expression of FERMT2 in human chorionic villi throughout gestation and determined the role of this integrin activator in trophoblast-substrate adhesion and invasion.

ITGA6/ITGB4 and ITGA6/ITGB1 are laminin binding integrin receptors in epithelia [51]. ITGA6/ITGB4 is highly expressed in CT, having been detected at both some lateral and primarily basal CT surfaces, while detected at lower levels in proximal EVT columns within pericellular regions [51]. Low levels of ITGB1 are also expressed in CT [13, 52, 53]. Thus, with co-localization of FERMT2 and ITGA6 in basal CT, FERMT2 is likely activating ITGA6/ITGB4 and ITGA6/ITGB1 for laminin binding to produce stable trophoblast-basement membrane adhesion [49, 54]. Depletion of FERMT2 in HTR8-SVneo trophoblast cells with specific siRNAs significantly decreased cell-substrate adhesion after 48 h, thus confirming such a role for this FERMT protein. Stable cell-substrate adhesion is also important for cell viability and siRNA-mediated FERMT2 depletion in HTR8-SVneo cells resulted in slightly reduced cell proliferation by day 3 post-transfection compared to non-targeting control cells. The statistically insignificant decrease in proliferation of FERMT2-depleted cells prior to day 3 post-transfection may reflect a subtle effect of the FERMT2 suppression on proliferation and the time required for any impact to be observed. In contrast, the lack of effect of FERMT2 depletion on proliferation observed at day 4 is likely a result of reduced control cell growth due to space limitations allowing the proliferation of FERMT2-depleted cells to recover by comparison.

Cell to cell adhesions are particularly prominent in chorionic villi (e.g. CT-CT or CT-syncytiotrophoblast) [49]. CDH1 is detected circumferentially around the CT and markedly decreases with differentiation and fusion of CT to syncytiotrophoblast [9, 10, 55]. FERMTs can be localized to cell-cell adhesions, particularly FERMT2 where it regulates the cytoskeleton [56–58]. Thus, the demonstrated co-localization of CDH1 with FERMT2 in this study indicates such signaling could have implications for CT differentiation as FERMT2 has been reported to promote muscle cell fusion in an integrin-dependent manner [56, 57, 59].

In our study, FERMT2 was localized to endothelial cells of developing capillaries and blood vessels within chorionic villi as indicated by the co-localization of FERMT2 with VWF. FERMT2 expression was also prevalent in mesenchyme around these vessels. FERMT2 expression has been detected in endothelial cells, with a role promoting integrin-mediated adhesion and migration, as well as in endothelial cell junctions promoting vascular barrier integrity [60, 61]. Further study is clearly required to understand the role of FERMT2 in placental vessel development.

The detection of FERMT2 throughout the EVT columns and particularly co-localization with ITGA5 in these cells, indicates a role in trophoblast migration and invasion. The trophoblast columns express ITGA6/ITGB4 in the proximal EVT, followed by more distal EVT cells expressing ITGA1/ITGB1, ITGA3/ITGB1, and ITGA5/ITGB1 [13, 53]. FERMT2 readily binds ITGB1 cytoplasmic domains and has also been reported to be highly expressed within cancers where it promotes migration and invasion [21]. Invasion assays in our study demonstrated that FERMT2 siRNA-mediated depletion in HTR8-SVneo cells significantly decreased trophoblast invasion. Impaired integrin activation occurs upon specific FERMT member knockout or knockdown phenotypes [62]. Thus, FERMT2 is likely an important regulator of ITGA1/ITGB1, ITGA3/ITGB1, and ITGA5/ITGB1 activation in EVT cells to stimulate migration and invasion. Montanez et al. [29] reported that FERMT2 homozygous null mice displayed peri-implantation lethality at embryonic day 7.5 due to a loss of integrin activation and epiblast detachment from the basement membrane. Thus, FERMT2 regulation of integrin-mediated signaling is conserved in mammals with hemochorial placentation and is likely very important for trophoblast differentiation.

It is important to acknowledge in both adhesion and invasion assays, that FERMT2 depletion did not result in loss of the majority of adhesion or invasion ability. We cannot rule out the possibility that FERMT1 can functionally compensate, at least in part, for the depletion of FERMT2. FERMT1 expression was unaffected and readily detected in HTR8-SVneo cells following FERMT2 depletion. Furthermore, all of the FERMTs, in concert with talin, can activate integrins by eliciting conformational changes in these receptors leading to increased integrin affinity for their extracellular ligands and regulating integrin-mediated adhesion and signaling [28–31]. FERMT1 expression and its role(s) in trophoblast behavior is currently being investigated.

It has been previously demonstrated that PTK2 and ILK are both highly expressed in human CT and EVT during gestation, where ILK can regulate syncytialization and trophoblast migration while PTK2 can control EVT migration and invasion [34, 35, 37, 63]. ILK and PTK2 are present at integrin clusters known as focal adhesions and can interact with the FERMT family of proteins [20, 21]. Recently, Huet-Calderwood et al. [62] demonstrated that FERMT2-mediated integrin activation required binding to an ILK-PINCH-Parvin complex. In the future, we will identify the integrin-FERMT interactome in trophoblast in order to clearly understand the precise regulation and implications of integrin-mediated signaling in the control of CT and EVT differentiation.

## Conclusions

While the switch in integrin expression within human villous and extravillous trophoblast during differentiation has been documented for quite some time, our findings are the first to identify in detail the presence of the FERMT2 integrin activator in human villous and extravillous trophoblast cells in situ. Furthermore, we have demonstrated the importance of FERMT2 in modulating trophoblast-substrate attachment and invasion in vitro. Thus, this adapter protein could directly influence integrin activation and subsequent integrin-mediated signaling that underlies trophoblast-substrate adhesion and invasion in vivo.

## Abbreviations

CDH1: E-cadherin
CT: Cytotrophoblast
DAPI: 4′,6-diamidino-2-phenylindole
ECM: Extracellular matrix
EVT: Extravillous trophoblast
FBLIM1: Migfilin
FBS: Fetal bovine serum
FERMT: Fermitin
FERMT1: Fermitin family homolog-1
FERMT2: Fermitin family homolog-2
IgG: Immunoglobulin
ILK: Integrin-linked kinase
ITGA1: Integrin alpha 1
ITGA6: Integrin alpha 6
ITGB1: Integrin beta 1
ITGB4: Integrin beta 4
MTT: 3-(4,5-Dimethylthiazol-2-yl)-2,5-diphenyltetrazolium bromide
PAGE: Polyacrylamide gel electrophoresis
PBS: Phosphate-buffered saline
PTK2: Focal adhesion kinase
SDS: Sodium dodecyl sulphate
siRNAs: Small interfering ribonucleic acids
TBST: Tris-buffered saline Tween-20
TUBA: Tubulin
VWF: Von Willebrand factor
